# Use of a non-probabilistic online panel as a control group for case–control studies to investigate food and waterborne outbreaks in Lower Saxony, Germany

**DOI:** 10.1017/S0950268821002594

**Published:** 2022-01-07

**Authors:** Delphine Perriat, Elke Mertens, Johannes Dreesman

**Affiliations:** 1 European Programme for Intervention Epidemiology Training (EPIET), European Centre for Disease Control and Prevention (ECDC), Solna, Sweden; 2 Department for Infectious Disease Epidemiology, Public Health Agency of Lower Saxony, Hannover, Germany

**Keywords:** Epidemiological study design, case-control studies, outbreaks, gastrointestinal infection, online panel

## Abstract

Established methods of recruiting population controls for case–control studies in infectious disease outbreak investigations are resource- and time-intensive, and are often subject to bias. The online panel have recently gained interest as an easy and timely method to select controls. We examined the feasibility, suitability and reliability of using an online panel to select controls for case–control studies as part of investigations of diffuse food and waterborne outbreaks. In January 2019, we deployed a web survey by email to the 277 members of a non-probabilistic online panel in Lower Saxony, Germany. We questioned them on basic sociodemographic characteristics and eating habits. They were frequency matched to cases on sex and age. Their food exposures were compared to those of traditionally recruited controls of four historical case–controls studies, which successfully investigated food and waterborne outbreaks. We used logistic regressions to assess the association between the food exposures and the disease (odds ratios). The use of a control panel successfully led to the identification of the food items in three of the four historical outbreak investigations, and their recruitment benefitted from increased speed and limited costs. Timely outbreak investigations would enable rapidly implementing control measures. We recommend the further evaluation of using panellists as controls in parallel case–control studies and case–panel studies.

## Introduction

In Germany, infectious disease outbreaks continue to pose a high burden on public health [1]. Control measures should be timely and rapidly implemented in order to prevent new infections and protect the health of the public. To decide on the best control measures, it is key to identify the outbreak source. Case–control methodologies are commonly used to achieve this [2]. Outbreak cases and controls are questioned about possible risk factors, and their answers are compared (e.g. food exposures during foodborne outbreaks).

Established methods of recruiting controls include case- or physician-nominated controls, random or sequential digit dialling and convenience sampling [2–4]. In addition of being resource-intensive, such methods are mostly slow, resulting in delays in identifying the source of infection, which can be vital for stopping severe outbreaks [5]. Additionally, those methods can be subject to selection bias, as controls are not representative of the population at risk.

New and innovative methods are required, which are easy and timely to implement and reduce bias [6]. Web surveys are increasingly used to collect data, including in the field of health and in epidemiological surveys as they are cost-effective while maintaining scientific rigour [7–10]. Moreover, methods that allow prior recruitment of controls are promising, because they offer the possibility to improve the timeliness and the representativeness of controls for the population that gave rise to the cases [11].

Recently, commercial online panels have shown encouraging results [12–14]. Those panels comprise individuals who have elected to receive internet-based questionnaires and can opt to complete them, usually in return of a reward. They are frequently used by marketing companies or polling organisations to obtain information about a target audience. Commercial online panels have successfully been used to collect epidemiological exposure data in retrospective [15] and prospective case–control studies [16–21]. One of the key benefits of this approach is that it allows efficiently and rapidly recruiting many controls, therefore enabling timely investigation of outbreaks.

According to German law, the primary responsibility of outbreak investigations lies with the local health authorities, and the coordination can be transferred to the state or national health authorities in complex situations (e.g. when the outbreak spread to several localities or regions) [22]. Analytic studies are a key component of outbreak investigations [23]. However, they are often not conducted as they require human and financial resources beyond typical case investigation and case finding efforts [24]. Local health authorities would highly benefit from the availability of online panels to recruit controls. However, such a method remains beyond their reach because of their limited public health resources. As part of efforts to strengthen the investigation of food and waterborne outbreaks of infectious gastrointestinal diseases at the local level, we identified the need to explore the use of an online panel as a source of controls for case–control studies as part of outbreak investigations.

The ‘Hygiene and Health Online Survey (HuGO)’ panel consists of 277 adults, who live in the federal state of Lower Saxony, Germany. They accepted to regularly answer online to health- and hygiene-related questions. We examined the feasibility, suitability and reliability of using the non-probabilistic online HuGO panel as a control group to recruit controls for case–control studies to investigate food and waterborne outbreaks in Lower Saxony, Germany.

## Methods

The data sources and data analysis steps are summarised in Table 1.

**Table 1.** Overview of the study methods to investigate the feasibility, suitability and reliability of using the Hygiene and Health Online Survey (HuGO) panel as a control group for case–control studies to investigate food and waterborne outbreaks in Lower Saxony, Germany, 2019

Data sources		Data analysis
**Lower Saxony Microcensus 2018:** population sample, which is considered representative of Lower Saxony (*n* = 6537)	------------------------------------------------------------------------------------------------------------------------→	**Suitability**: Comparison of the sociodemographic characteristics of the HuGO panellists with participants to the Lower Saxony Microcensus 2018 (chi2 goodness of fit test).
**Hygiene and Health Online Survey (HuGO) panel:** non-probabilistic online panel comprised of 277 adults living in Lower Saxony, Germany, who accepted to answer hygiene- and health-related questions, 2019	------------------------------------------------------------------------------------------------------------------------→	**Feasibility**: Computation of response rates among HuGO panellists, and description of the human and financial resources required to conduct the HuGO and historical studies.
**Historical studies:** four case–control studies that successfully investigated food and waterborne outbreaks in Lower Saxony and neighbouring federal states in Germany, 2001–2017	------------------------------------------------------------------------------------------------------------------------→	**Reliability**: Comparison of the strength of the association (using odds ratios) between food and water exposures and disease, using the four ‘historical studies’ (case–control studies with historical cases and historical controls) vs. the four ‘HuGO studies’ (case–control studies with historical cases and HuGO panellists as controls).

### Data sources

We used three data sources:

- The Lower Saxony Microcensus 2018: The Microcensus contains basic sociodemographic information on a randomly selected sample of the population living in Lower Saxony, Germany (*n* = 6537), including gender, nationality, age, household composition, school and postgraduate training and employment status. This sample is considered representative of Lower Saxony [25].
- Four historical case–control studies: The four historical studies successfully investigated food and waterborne outbreaks in Lower Saxony and in the neighbouring German federal States between 2001 and 2017, using case–control designs: *Campylobacter jejuni* infection via tap water (study A), *Salmonella enterica Bovismobificans* infection via raw pork (study B), *Salmonella enterica Goldcoast* infection via raw pork (study C) and *Salmonella enterica Oranienburg* via chocolate (study D). Details of the conduction of the studies are given in Table 2. Parallel microbiological investigations to the studies provided microbiological evidence that supported the epidemiological findings.
- The Hygiene and Health Online Survey (HuGO) panel: To form the HuGO panel, participants from the Hygiene and Behaviour Infectious Diseases Study (HaBIDS) panel were integrated. The HaBIDS panel was a population-based longitudinal panel, created by the Helmholtz Centre for Infection Research in 2014. A total of 2.379 participants aged 15 to 69 years were drawn by means of probability sampling from the regional population registries of Lower Saxony [26]. They were regularly consulted to answer online to questions on knowledge, attitudes and practices related to infections in Lower Saxony. In 2015, the HaBIDS panel included 1.037 participants aged more than 18 years old [27]. In 2018, they were offered to form the HuGO panel, led by the Public Health Agency of Lower Saxony (Niedersächsiches Landesgesundheitsamt, NLGA, https://www.nlga.niedersachsen.de/). The panel aims at supporting the activities of the Public Health Agency in infection prevention and control. This includes the investigation of outbreaks and the quick assessment of the population perceptions towards infection control measures. A total of 277 members of the HaBIDS panel agreed to form the HuGO panel. On 17 January 2019, the HuGO panellists were asked by email to answer an online survey, in which they were questioned about their sociodemographic characteristics and eating habits of the past week. The sociodemographic questions were based on the Lower Saxony Microcensus 2018, and the eating habit questions were based on the four historical case–control studies. A reminder was sent on 22 February 2019. As controls of case–control study should be free of the outcome of interest, panellists with gastrointestinal symptoms in the week prior to answering the questionnaire were filtered out using a dedicated question at the beginning of the online questionnaire.

**Table 2.** Key features of four historical case–control studies that successfully investigated food and waterborne outbreaks in Germany between 2001 and 2017

Study identifier	A	B	C	D
Study period	July 2017	February 2005	June 2004	September 2001
Study area	City of Lamspringe, Lower Saxony	Lower Saxony	Saxony Anhalt (neighbouring state of Lower Saxony)	Several German states (including Lower Saxony)
Hypothesised exposure	Consumption of contaminated tap water	Consumption of contaminated raw pork	Consumption of contaminated raw pork	Consumption of contaminated chocolate
Pathogen (microbiological investigation)	*Campylobacter jejuni*	*Salmonella enterica Bovismobificans*	*Salmonella enterica Goldcoast*	*Salmonella enterica Oranienburg*
Control selection	Random digital dialling among inhabitants of Lamspringe	Random digital dialling among inhabitants of Lower Saxony	Random digital dialling among inhabitants of Saxony Anhalt	Random digital dialling among inhabitants of Germany, with 1:1 individual matching on sex and age
Data collection	Telephone interviews conducted by 4 staff members of the state health authority during 7 days. Overall 111 persons were called; 46 accepted to participate (41% response rate), among which 35 met the control selection criteria (e.g. presence in Lamspringe during the outbreak period)	Telephone interviews	Telephone interviews	Telephone interviews conducted by a large team of local, state and national health agency staff
Number of controls [Number of cases]	35 [12]	37 [38]	54 [14]	50 [48]
Studied exposures	• sex, age • consumption in the last 7 days of unboiled tap water at home, unboiled tap water outside of home	• sex, age • consumption in the last 7 days of raw pork, raw sausage pork, cooked pork, raw beef, raw egg, salad, sprout	• sex, age • consumption in the last 3 days of raw pork, raw sausage pork, cooked pork, raw beef, raw egg	• sex, age • shopping in the last 7 days in the supermarket chain X • consumption in the past 7 days of chocolate bought in the supermarket chain X
Available data source	Individual-level data	Individual-level data	Aggregated data	Aggregated data
Outbreak details	A heavy rainfall led to a contamination of water supplies with surface water in the city of Lamspringe. Interviews of controls were conducted around 14 days after the first cases developed symptoms. At that time, information related to the likely contaminated tap water was communicated in the newspapers, with a recommendation to drink bottled water.		In 2004, Lower Saxony and Saxony Anhalt reported most of the *S. Goldcoast* cases in Germany [39]. A majority of cases included in the case–control study reported having eaten raw pork, which was found to have been supplied by a slaughterhouse in Lower Saxony. *S. Goldcoast* was found in raw pork as part of self-checks in this slaughterhouse. In Lower Saxony, over 90% of the surveyed cases stated that they had eaten raw pork products in the 3 days before disease onset.	A trace-back analysis of the implicated chocolates along supply chains allowed to identify that they were contaminated prior to their distribution in Germany by the supermarket chain X, and in other European countries [40]

### Data analysis

We explored the use of the panel according to three components:

- **Feasibility of using the HuGO panel as a control group in outbreak investigations.** We reported the human and financial resources required to conduct the case–control study using panellists as controls, including the creation of the online questionnaire. We reported the response rate among HuGO panellists after receiving the questionnaire: on the same day, after one day, after one week, after receiving a reminder, and in total. When available, we provided information on the time, human and financial resources required to conduct the historical studies.
- **Suitability of the HuGO panel for Lower Saxony in terms of basic sociodemographic characteristics.** We compared the panellists to the sample of the Lower Saxony Microcensus 2018 on sex, age, nationality, household composition, education level and employment status, using chi2 goodness of fit tests.
- **Reliability of using the HuGO panel as a control group in the investigation of four historical outbreaks.** We refer as ‘historical studies’ to case–control studies with historical cases and historical controls, and as ‘HuGO studies’ to case–control studies, with historical cases and HuGO panellists as controls. In the HuGO studies, we controlled for the possible confounding effects of sex and age by matching the frequencies between historical cases and panellists on both variables (Supplementary File 1). When the information on sex or age of historical cases was not available, the frequencies of panellists were matched to those of the Lower Saxony Microcensus 2018. Separately in historical and in HuGO studies, we used univariable logistic regression analyses to assess the association between the food and water exposures and the disease. When possible, we performed multivariable logistic regressions. We included in the regression models all exposure variables that were associated with the disease with a *p*-value less than 0.2 in univariable analyses. We also included in the models the categorical variables for age and sex (as possible confounders) when the controls where not frequency matched to cases on those variables. Odds ratios (OR) were used to determine whether a particular exposure was a risk factor for the occurrence of the disease. The 95% confidence interval (95% CI) was used to estimate the precision of the OR. An exposure was regarded as a risk factor if the OR was >1 and the *p*-value <0.05, or as a protective factor if the OR was <1 and the *p*-value <0.05. If an exposure was a risk factor or a protective factor in both historical and HuGO studies, we considered that both studies had similar results.

All analyses were performed using the statistical software R.

## Results

### Feasibility

One scientist, working for the Public Health Agency of Lower Saxony conducted the study. A working day was required to create the online questionnaire using the Lamapoll® software. Apart from the software cost, no additional financial resource was required. A total of 203/277 (73%) HuGO panellists answered the survey: 76/277 (27%) answered the questionnaire on the day they received it, adding up to 107/277 (39%) on the following day and to 152/277 (54%) within a week. After receiving a reminder, a month later, 30/277 (11%) additional panellists answered.

### Suitability

There were statistically significant differences in the distribution of all measured sociodemographic characteristics between the panellists and the population of Lower Saxony (Table 3). For example, the proportion of women participating in the HuGO panel was higher than in the population of Lower Saxony (63% *vs.* 51%), the proportion of people younger than 30 was lower (7% *vs.* 17%) and a higher proportion attended university (32% *vs.* 9%).

**Table 3.** Comparison of sociodemographic characteristics between the Hygiene and Health Online Survey (HuGO) panellists (*n* = 203) and the population of Lower Saxony (Lower Saxony Microcensus 2018)

	HuGO-Panel (*n* = 203)		Lower Saxony Microcensus (*n* = 6537)		*χ*^2^ goodness of fit test
	*n*	%	*n*	%	*p-*value
Sex					
male	74	37	3232	49	
female	129	63	3304	51	<0.001
Nationality					
German	201	99	5956	91	
not German	2	1	581	9	<0.001
Age group					
≥18 and <30	14	7	1110	17	
≥30 and <40	24	12	938	14	
≥40 and <50	41	20	1054	16	
≥50 and <60	73	36	1286	20	
≥60	51	25	2148	33	<0.001
Household					
lives alone	29	14	1630	25	
≥2 persons	174	86	4907	75	<0.001
Education level					
no post-secondary education^1^	5	2	1612	25	
apprenticeship^2^	80	39	3447	53	
technical college^3^	17	8	481	7	
university of applied science^4^	36	18	400	6	
university^5^	65	32	595	9	<0.001
Employement status					
has a job	147	72	3916	60	
does not have a job or is retired	56	28	2621	40	<0.001

German translations: ^1^kein Berufsabschluss, ^2^Lehre/Berufsausbildung, ^3^Fachschulabschluss, ^4^Fachhochschule/Berufsakademie, ^5^Universität / Promotion.

### Reliability

No panellists experienced gastrointestinal symptoms in the week prior to the survey. Table 4 summarises the results of the univariable logistic regression analyses for the food items that were identified as outbreak sources in the historical studies. Results of univariable analyses where controls were frequency matched to cases on sex and age, and results of multivariable analyses for all food items are provided in the Supplementary File 2.

**Table 4.** Comparison of the strength of association between food and water exposures and disease (odds ratios) in univariable univariable logistic regression analyses between historical and HuGO case–control studies to investigate food and waterborne outbreaks, Germany 2019

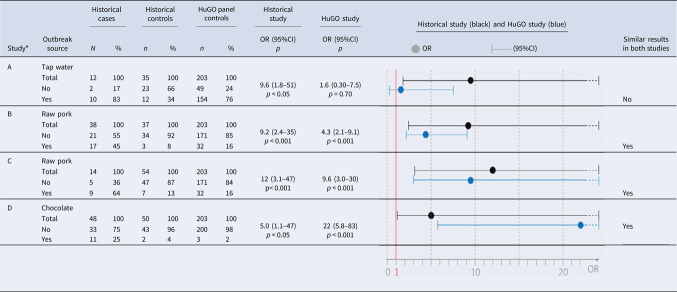

Historical study: case–control study with historical cases and historical controls, HuGO study: case–control study with historical cases and HuGO panel controls (2019), OR: Odds Ratio, 95% CI: 95% Confidence Interval, *p*: two-sided *p*-value from Fisher's exact test, *The four historical studies successfully investigated food and waterborne outbreaks in Lower Saxony and in the neighbouring German federal states, using case–control designs: *Campylobacter jejuni* infection via tap water in 2017 (study A), *Salmonella enterica Bovismobificans* infection via raw pork in 2005 (study B), *Salmonella enterica Goldcoast* infection via raw pork in 2004 (study C) and *Salmonella enterica Oranienburg* via chocolate in 2001 (study D).

Using panellists as controls to investigate the four historical outbreaks gave consistent results in three instances. In the studies B, C and D, the ORs of the outbreak sources were significant and of similar magnitude to those of the historical studies. In Study A, drinking tap water at home was associated with the disease in the historical study (Odds Ratio in univariable analysis (ORu) = 9.6 (1.8–51)), but not in the HuGO study (ORu = 1.6 (0.33–7.5)). Results were consistent in univariable and multivariable analyses, with or without frequency matching.

## Discussion

We investigated the feasibility, suitability and reliability of using panellists as controls in case–control studies to investigate food and waterborne outbreaks. We found that using panellists would lead to similar results as using traditional controls, and the study would benefit from increased speed of recruitment as well as limited costs for public health action.

## Timely and low-cost outbreak investigation thanks to panel controls

The majority of panellists responded to the online questionnaire in less than a week, ensuring a higher response rate than traditional methods such as random digit dialling [9, 13]. The recruitment of panellists as controls also required far less staff and financial resources than other approaches, as it amounts to sending an email rather than conducting numerous face-to-face or telephone interviews [12, 14]. In the event of an actual food or waterborne outbreak, the HuGO panel offers a more timely and cost-effective control recruitment and analysis, which would lead to timelier public health actions to limit additional cases (e.g. trace-back investigations, recall of products). It is nevertheless important to consider that the constitution of the HaBIDS panel, from which the HuGO panel stems, did require substantial resources. Indeed, the 2.379 panellists were recruited thanks to the dedication of a 1–2 researchers, who drew 16.000 people from the registries of Lower Saxony and send them invitation letters per post therefore reaching an overall response rate of 8.9% [26].

## Suitability of panel controls for outbreak investigation

The study assessed the suitability of panel controls for case–control studies. Ideally, one would be confident using panellists as controls if these were not more biased than controls used in current best practices. Controls are used to estimate the prevalence of exposures in the population that gave rise to the cases. They are expected to be free of the outcome of interest, representative of the population at risk of the outcome and selected independently of the exposure of interest [28]. In the situation of an outbreak investigation, case–control studies aim to estimate a particular exposure-disease association, while appropriately controlling for confounding and avoiding other biases.

Selection bias is a particular problem inherent in case–control studies, where it gives rise to non-comparability between cases and controls, threatening the generalisability of the study results [2]. Our study is affected by a sampling bias, as HuGO panellists volunteered to participate in the study. First, HuGO panellists differ from the general population of Lower Saxony in terms of sociodemographic factors such as age, sex and education level. We reduced this bias by matching the frequencies between historical cases and panellists on sex and age, therefore controlling for the possible confounding effects of both variables. Additionally, controls recruited through traditional methods are also rarely representative of the general population [2]. It is largely accepted that a lack of representativeness on socio-demographic characteristics does not hamper scientific inference [29, 30]. Valid scientific inference is achieved if the confounders are controlled for, and there is no reason to believe that control of confounding can be more easily achieved in a randomly selected control group that in a panel group [29]. Second HuGO panellists are significantly more educated than the general population of Lower Saxony. As studies have shown that a higher education status is associated with healthier eating habits [31], it is likely that HuGO panellists may be more health-conscious than the general population [7, 12].

Using the HuGO panellists as controls decreased the likelihood of information bias as compared to using traditional controls, resulting in a greater validity of the food exposure information. First, there is little recall bias as panellists responded within a few days to the questionnaire. As traditional controls are often recruited with days or weeks of delay after the outbreak occurs, they have more difficulties to remember their food exposures at a particular time point. Panellists would then be particularly useful when investigating outbreaks caused by uncommon food exposures, as they are oftentimes more forgotten than common food exposures [32].

Additionally, online surveys are less likely to suffer from social desirability bias as other data collection methods, such as phone or face-to-face interviews [26]. Panellists are less likely to underreport ‘bad’ food items, and over report ‘good’ food items than traditional controls. They would therefore also be particularly adequate to investigate sensitive exposures, benefitting from the survey anonymity [12, 26].

## Reliability of control panels to identify outbreak sources

The study explored the circumstances in which using panellists would be reliable to investigate food and waterborne outbreaks. Indeed, investigators expect consistent conclusions regarding the likely outbreak source, regardless of the study design they use for the investigation.

In the present study, three of four outbreak sources could be identified using panellists as controls (studies B, C and D). The differences in the magnitude of the effect estimates (odds ratio) between historical and HuGO studies did not affect the ability of panellists to successfully identify the outbreak sources. As odds ratios measuring associations between contaminated food items and the disease are usually very high during food and waterborne outbreaks [32], we argue that they are not significantly affected by differences in exposure proportions between panellists and traditional controls.

The study shows that in outbreak A, the association between the consumption of tap water and gastrointestinal disease could not be identified with a statistical significance by using panellists as controls. We hypothesise that, in this particular situation, panellists did not have the chance of being exposed to the very localised outbreak source (contamination of water supplies with surface water due to heavy rainfall), and were thus not representative of the population at risk of the outcome. They did not live in the same area as the cases, and were not questioned at the particular time during which the waterborne outbreak occurred. This result furthers the argument that the reliability of panellists is likely to be higher when used as controls in the investigation of region- or nationwide outbreaks (like outbreaks B, C and D) as compared to very local outbreaks (like outbreak A). On the other hand, historical controls were likely to have changed their drinking habits, and drank significantly less tap water than usual, as they were interviewed around 14 days after the first cases developed symptoms. At that time, information related to the contaminated tap water was already communicated in the news, with a recommendation to rather drink bottled water.

## Strengths and limitations

The main strength of this study is that it is a proof of concept for a promising method to recruit controls for case–control studies. Panellists can be asked specific questions about particular products they ate, ways of cooking, places they shopped at etc., as soon as an outbreak occurs, enabling a quick investigation. The present study participates in the efforts to provide local public health professionals with innovative methods to empower them in the conduction of outbreak investigations [33]. The study also assesses likely bias of using panellists as controls in the context of outbreak investigations. It supports the findings of other studies that the risk of bias must be assessed anew when a study is conducted to investigate an outbreak [11]. Yet overall, the risk of bias when using the HuGO panel should be smaller in situations where the cases seem to resemble the general population (with a tendency of higher education because this is what the panel is composed of). Another strength of the study is the oversampling of the control group. This allows the selection of a subset of more valid controls to match the frequencies of cases in terms of sex and age, thereby addressing confounding and some of the bias introduced by using panellists as controls. Such an approach is here possible given that there is no additional cost per additional questionnaire.

The main limitation of the study concerns the historical studies. Panellists could only be used when population-based controls were used in the historical studies, but not in specific settings (e.g. outbreaks during a party or at a restaurant). This strongly reduced the choice of historical studies for this analysis. The available studies had limited information on some key exposures and multivariable analyses could therefore not always be conducted. Additionally, the panel is affected by a selection bias. As this cannot be controlled via study design, it is therefore important to consider whether the measure of effect for a particular exposure may have been due to such an inherent sampling issue. In the situation of a prospective outbreak investigation, socio-demographic data could easily be collected among cases and panellists, and a weighting method could minimise this bias (e.g. frequency matching, propensity scores, quotas) [13].

## Perspectives

The study provides encouraging results and warrant further exploration to prove the validity of using panellists as controls in case–control studies. First, we will recruit more panellists (including children), in order to increase the sociodemographic diversity of the panel and its suitability to investigate the upcoming outbreaks. Then, as soon as a likely food or waterborne outbreak occurs in Lower Saxony, we will conduct two parallel case–control studies in which controls would be selected either through a traditional method or from the HuGO panel. The results of both studies will be compared. Propensity score matching will be used to reduce selection bias [34, 35].

Finally, in order to assess in which scenarios or for which hypothesised food and water exposures or behaviours using panellists as controls might be more appropriate than traditional controls, any selection bias introduced by using the panel need to be better understood. A prospect is to compare the reported food and water exposures and behaviours of panellists with that of probability samples from population-based food exposure surveys, such as with the German food exposure survey [36, 37] or other sources of such data for which selection biases are minimised or previously characterised [38].

## Conclusion

The study shows that using panellists as controls in case–control studies is feasible and suitable to investigate diffuse outbreaks within an adequate time frame, and researchers can benefit from increased speed of recruitment and limited costs. Nevertheless, the circumstances in which panellists are reliable to investigate food and waterborne outbreaks should be further investigated. We therefore recommend the further evaluation of this approach in parallel case–control studies and case-panel studies, especially in the context of food and waterborne outbreak investigations.

## Data Availability

Data supporting the findings of this study are openly available in Zenodo at https://doi.org/10.5281/zenodo.5243412, reference number [5243412].

## Supplementary material

For supplementary material accompanying this paper visit http://doi.org/10.1017/S0950268821002594. click here to view supplementary material
