# Supplementary material for: Use of a non-probabilistic online panel as a control group for case–control studies to investigate food and waterborne outbreaks in Lower Saxony, Germany
Source: Epidemiol Infect. 2022 Jan 7;150:e53. doi: 10.1017/S0950268821002594 (PMC8915193; doi:10.1017/S0950268821002594)
Supplement: Supplementary file 1 [file hygsup.zip › S0950268821002594sup001.docx]

**Supplementary File 1**

**Table 1: Study A. Frequency Matching on age and sex in the HuGO study (historical cases and HuGO panel controls), using individual-level data for historical cases, for the investigation of a *Campylobacter jejuni* outbreak via tap water in Germany, 2019 (study A)**

|  | Female | | | Male | | |
| --- | --- | --- | --- | --- | --- | --- |
|  | historical cases | panel controls | panel controls, FM | historical cases | panel controls | panel controls, FM |
| ≥ 18 and <30 | 0 | 9 | 0 | 1 | 5 | 5 |
| ≥ 30 and <40 | 0 | 16 | 0 | 0 | 8 | 0 |
| ≥ 40 and <50 | 1 | 28 | 5 | 1 | 13 | 5 |
| ≥ 50 and <60 | 2 | 51 | 10 | 2 | 22 | 10 |
| ≥ 60 | 2 | 25 | 10 | 3 | 26 | 15 |
| total | 5 | 129 | 25 | 7 | 74 | 35 |

FM: Panel controls were randomly selected to reach a case:control ratio of 1:5 in each age-sex category. Details on the historical study are provided in the Table 2 of the manuscript.

**Table 2: Study B. Frequency Matching on age and sex in the HuGO study (historical cases and HuGO panel controls), using individual-level data for historical cases, for the investigation of a *Salmonella enterica Bovismobificans* outbreak via raw pork in Germany, 2019 (study B)**

|  | Female | | | Male | | |
| --- | --- | --- | --- | --- | --- | --- |
|  | historical cases | panel controls | panel controls, FM | historical cases | panel controls | panel controls, FM* |
| ≥ 18 and <30 | 0 | 9 | 0 | 1 | 5 | 3 |
| ≥ 30 and <40 | 4 | 16 | 12 | 1 | 8 | 3 |
| ≥ 40 and <50 | 4 | 28 | 12 | 5 | 13 | 13 |
| ≥ 50 and <60 | 2 | 51 | 6 | 5 | 22 | 15 |
| ≥ 60 | 7 | 25 | 21 | 9 | 26 | 26 |
| total | 17 | 129 | 51 | 21 | 74 | 60 |

FM: Panel controls were randomly selected to reach a case:control ratio of 1:3 in each age-sex category., *Two controls are missing among men in the age category ≥ 40 and <50 to reach the case:control ratio 1:3, and One control is missing among men in the age category ≥ 60 to reach the case:control ratio 1:3. Details on the historical study are provided in the Table 2 of the manuscript.

**Table 3: Study C. Frequency Matching on age and sex in the HuGO study (historical cases and HuGO panel controls), using aggregated data from the Lower Saxony Microcensus, for the investigation of a *Salmonella enterica Goldcoast* outbreak via raw pork in Germany, 2019 (study C)**

|  | Female | | | Male | | |
| --- | --- | --- | --- | --- | --- | --- |
|  | Lower Saxony Microcensus  (%) | panel controls | panel controls, FM | Lower Saxony Microcensus % | panel controls | panel controls, FM |
| ≥ 18 and <30 | 16 | 9 | 9 | 18 | 5 | 5 |
| ≥ 30 and <40 | 14 | 16 | 8 | 14 | 8 | 4 |
| ≥ 40 and <50 | 16 | 28 | 9 | 17 | 13 | 5 |
| ≥ 50 and <60 | 20 | 51 | 11 | 20 | 22 | 6 |
| ≥ 60 | 35 | 25 | 20 | 31 | 26 | 9 |
| total | 100 | 129 | 57 | 100 | 74 | 29 |

FM: Panel controls were randomly selected to reproduce the distribution of sex and age of the population of Lower Saxony. Details on the historical study are provided in the Table 2 of the manuscript.

**Table 4: Study D. Frequency Matching on sex in the HuGO study (historical cases and HuGO panel controls), using aggregated data for historical cases, for the investigation of a *Salmonella enterica Oranienburg* outbreak via chocolate in Germany, 2019 (study D)**

|  | Female | | | Male | | |
| --- | --- | --- | --- | --- | --- | --- |
|  | historical cases | panel controls | panel controls, FM | historical cases | panel controls | panel controls, FM |
| total | 24 | 129 | 74 | 24 | 74 | 74 |

FM: Panel controls were randomly selected to reach a case:control ratio of 1:3 in each age-sex category. Details on the historical study are provided in the Table 2 of the manuscript.
