# Supplementary material for: Use of a non-probabilistic online panel as a control group for case–control studies to investigate food and waterborne outbreaks in Lower Saxony, Germany
Source: Epidemiol Infect. 2022 Jan 7;150:e53. doi: 10.1017/S0950268821002594 (PMC8915193; doi:10.1017/S0950268821002594)
Supplement: Supplementary file 1 [file hygsup.zip › S0950268821002594sup002.docx]

**Supplementary File 2**

**Table 1. Univariable logistic regression analyses of sex, age and water exposures for gastrointestinal disease in historical and HuGO case-control studies for the investigation of a *Campylobacter jejuni* outbreak via tap water, Germany, 2019 (study A)**

|  | historical cases | | historical controls | | panel controls | | panel controls, FM | | historical study | | HuGO study | | HuGO study, FM | |
| --- | --- | --- | --- | --- | --- | --- | --- | --- | --- | --- | --- | --- | --- | --- |
|  | n | % | n | % | n | % | n | % | OR [95%CI] | p | OR [95%CI] | p | OR [95%CI] | p |
| **total** | 12 | 100 | 35 | 100 | 203 | 100 | 60 | 100 |  |  |  |  |  |  |
| **sex*** |  |  |  |  |  |  |  |  |  |  |  |  |  |  |
| male | 7 | 58 | 9 | 28 | 74 | 36 | 35 | 58 |  |  |  |  |  |  |
| female | 5 | 42 | 23 | 72 | 129 | 64 | 25 | 42 | 0.27 [0.07-1.1] | 0.08 | 0.40 [0.12-1.3] | 0.14 | - | - |
| **age group**** |  |  |  |  |  |  |  |  |  |  |  |  |  |  |
| >18 & ≤55 | 6 | 50 | 5 | 14 | 118 | 58 | 28 | 47 |  |  |  |  |  |  |
| >55 | 6 | 50 | 29 | 86 | 85 | 42 | 32 | 53 | 0.16 [0.03-0.72] | < 0.05 | 1.4 [0.43-4.4] | 0.76 | - | - |
| **has drunk unboiled tap water at home** |  |  |  |  |  |  |  |  |  |  |  |  |  |  |
| no | 2 | 17 | 23 | 66 | 49 | 24 | 17 | 28 |  |  |  |  |  |  |
| yes | 10 | 83 | 12 | 34 | 154 | 76 | 43 | 72 | 9.6 [1.8-51] | < 0.05 | 1.6 [0.33-7.5] | 0.73 | 1.9 [0.39-9.9] | 0.49 |
| **has drunk unboiled tap water outside of home** |  |  |  |  |  |  |  |  |  |  |  |  |  |  |
| no | 10 | 83 | 33 | 94 | 141 | 70 | 46 | 77 |  |  |  |  |  |  |
| yes | 2 | 17 | 2 | 6 | 62 | 30 | 14 | 23 | 3.3 [0.41-26] | 0.26 | 0.45 [0.10-2.1] | 0.51 | 0.72 [0.14-3.7] | 1.0 |

historical study: case-control study with historical cases and historical controls (2017), HuGO study: case-control study with historical cases and HuGO panel controls (2019), FM: the frequencies of controls are matched to the frequencies of cases on sex and age (case:control ratio 1:5, 5 age categories: ≥18 and <30, ≥30 and <40, ≥40 and <50, ≥50 and <60, ≥60), OR: Odds Ratio, 95%CI: 95% Confidence Interval, p: two-sided p-value from Fisher's Exact test, -: not applicable, *Information on sex is missing for 3 historical controls, **Information on age is missing for 1 historical control. Details on the historical study are provided in the Table 2 of the manuscript.

**Table 2. Multivariable logistic regression analysis of exposures for gastrointestinal disease in the historical case-control study for the investigation of a *Campylobacter jejuni* outbreak via tap water, Germany, 2017 (study A)**

|  | OR [95%CI] | p |
| --- | --- | --- |
| **historical study** |  |  |
| **has drunk unboiled tap water at home** | **11 [2.4-120]** | **<0.05** |
| age >55 years old | 0.15 [0.01-0.64] | 0.03 |
| female sex | 0.32 [0.07-1.1] | 0.08 |

OR: Odds Ratio, 95%CI: 95% Confidence Interval, p: p-value. Details on the historical study are provided in the Table 2 of the manuscript.

**Table 3. Univariable logistic regression analyses of sex, age and food exposures for gastrointestinal disease in historical and HuGO case-control studies for the investigation of a *Salmonella enterica Bovismobificans* infection via raw pork, Germany, 2019 (study B)**

|  | historical cases | | historical controls | | panel controls | | panel controls, FM | | historical study | | HuGO study | | HuGO study, FM | |
| --- | --- | --- | --- | --- | --- | --- | --- | --- | --- | --- | --- | --- | --- | --- |
|  | n | % | n | % | n | % | n | % | OR [95%CI] | p | OR [95%CI] | p | OR [95%CI] | p |
| **total** | 38 | 100 | 37 | 100 | 203 | 100 | 111 | 100 |  |  |  |  |  |  |
| **sex*** |  |  |  |  |  |  |  |  |  |  |  |  |  |  |
| male | 21 | 55 | - | - | 74 | 37 | 60 | 55 |  |  |  |  |  |  |
| female | 17 | 45 | - | - | 129 | 63 | 51 | 45 | - | - | 0.46 [0.23-0.94] | < 0.05 | - | - |
| **age group*** |  |  |  |  |  |  |  |  |  |  |  |  |  |  |
| >18 & <57 | 19 | 50 | - | - | 135 | 67 | 63 | 57 |  |  |  |  |  |  |
| ≥57 | 19 | 50 | - | - | 68 | 33 | 48 | 43 | - | - | 1.9 [0.98-3.9] | 0.06 | - | - |
| **raw pork** |  |  |  |  |  |  |  |  |  |  |  |  |  |  |
| no | 21 | 55 | 34 | 92 | 171 | 85 | 93 | 84 |  |  |  |  |  |  |
| yes | 17 | 45 | 3 | 8 | 32 | 16 | 18 | 16 | **9.2 [2.4-35]** | **< 0.001** | **4.3 [2.1-9.1]** | **< 0.001** | **4.2 [1.8-9.4]** | **< 0.05** |
| **raw pork (sausage)** |  |  |  |  |  |  |  |  |  |  |  |  |  |  |
| no | 24 | 63 | 21 | 57 | 136 | 67 | 65 | 59 |  |  |  |  |  |  |
| yes | 14 | 37 | 16 | 43 | 67 | 33 | 46 | 41 | 0.77 [0.30-1.9] | 0.6 | 1.2 [0.58- 2.4] | 0.70 | 0.82 [0.38-1.7] | 0.70 |
| **cooked pork** |  |  |  |  |  |  |  |  |  |  |  |  |  |  |
| no | 27 | 71 | 26 | 70 | 115 | 57 | 63 | 57 |  |  |  |  |  |  |
| yes | 11 | 29 | 11 | 30 | 88 | 43 | 48 | 43 | 0.96 [0.36-2.6] | 1.0 | 0.53 [0.25-1.1] | 0.10 | 0.53 [0.24-1.2] | 0.13 |
| **raw beef** |  |  |  |  |  |  |  |  |  |  |  |  |  |  |
| no | 38 | 100 | 36 | 97 | 200 | 98 | 109 | 98 |  |  |  |  |  |  |
| yes | 0 | 0 | 1 | 3 | 3 | 2 | 2 | 2 | 0 | 1.0 | 0 | 1.0 | 0 | 1.0 |
| **raw egg** |  |  |  |  |  |  |  |  |  |  |  |  |  |  |
| no | 38 | 100 | 37 | 100 | 198 | 97 | 109 | 98 |  |  |  |  |  |  |
| yes | 0 | 0 | 0 | 0 | 5 | 23 | 2 | 2 | 0 | 1.0 | 0 | 1.0 | 0 | 1.0 |
| **salad** |  |  |  |  |  |  |  |  |  |  |  |  |  |  |
| no | 37 | 97 | 33 | 89 | 96 | 47 | 56 | 50 |  |  |  |  |  |  |
| yes | 1 | 3 | 4 | 11 | 107 | 53 | 55 | 50 | 0.22 [0.02-2.1] | 0.2 | 0.02 [0-0.18] | < 0.001 | 0.03 [0-0.20] | <0.001 |
| **sprout** |  |  |  |  |  |  |  |  |  |  |  |  |  |  |
| no | 37 | 97 | 36 | 97 | 181 | 89 | 96 | 86 |  |  |  |  |  |  |
| yes | 1 | 3 | 1 | 3 | 22 | 11 | 15 | 14 | 0.97 [0.06-16] | 1.0 | 0.22 [0.03-1.7] | 0.14 | 0.17 [0.02-1.3] | 0.07 |

historical study: case-control study with historical cases and historical controls (2005), HuGO study: case-control study with historical cases and HuGO panel controls (2019), FM: the frequencies of controls are matched to the frequencies of cases on sex and age (case:control ratio: 1:3, 5 age categories: ≥ 18 and <30, ≥30 and <40, ≥40 and <50, ≥50 and<60, ≥60), OR: Odds Ratio, 95%CI: 95% Confidence Interval, p: two-sided p-value from Fisher's Exact test, -: not applicable, *Information on sex and age is missing for the historical controls. Details on the historical study are provided in the Table 2 of the manuscript.

**Table 4. Multivariable logistic regression analyses of exposures for gastrointestinal disease in historical and HuGO case-control studies for the investigation of a *Salmonella enterica Bovismobificans* infection via raw pork, Germany, 2019 (study B)**

|  | OR [95%CI] | p |
| --- | --- | --- |
| **historical study** |  |  |
| **raw pork** | **6.2 [2.3 - 24]** | **<0.001** |
| salad | 0.17 [0.01 – 0.93] | 0.07 |
| **HuGO study** |  |  |
| **raw pork** | **4.6 [2.4 – 9.8]** | **<0.001** |
| salad | 0.06 [0.007 - 0.18] | <0.001 |
| female sex | 0.57 [0.31 – 1.1] | 0.08 |
| cooked pork | 0.60 [0.30 - 1.2] | 0.13 |
| age >57 years old | 1.4 [0.78-2.7] | 0.23 |
| sprout | 0.51 [0.06 - 2.0] | 0.42 |
| **HuGO study,** **FM** |  |  |
| **raw pork** | **1.9 [1.1-3.3]** | **<0.05** |
| salad | 0.12 [0.02-0.38] | <0.05 |
| cooked pork | 0.75 [0.41-1.3] | 0.34 |
| sprout | 0.57 [0.07-2.1] | 0.46 |

historical study: case-control study with historical cases and historical controls (2005), HuGO study: case-control study with historical cases and HuGO panel controls (2019), FM: the frequencies of controls are matched to the frequencies of cases on sex and age (case:control ratio: 1:3, 5 age categories: ≥ 18 and <30, ≥30 and <40, ≥40 and <50, ≥50 and <60, ≥60), OR: Odds Ratio, 95%CI: 95% Confidence Interval, p: p-value. Details on the historical study are provided in the Table 2 of the manuscript.

**Table 5. Univariable logistic regression analyses of sex, age and food exposures for gastrointestinal disease in historical and HuGO case-control studies for the investigation of *Salmonella enterica Goldcoast* outbreak via raw pork, Germany, 2019 (study C)**

|  | historical cases | | historical controls | | panel controls | | panel controls, FM | | historical studies | | HuGO study | | HuGO study, FM | |
| --- | --- | --- | --- | --- | --- | --- | --- | --- | --- | --- | --- | --- | --- | --- |
|  | n | % | n | % | n | % | n | % | OR [95%CI] | p | OR [95%CI] | p | OR [95%CI] | p |
| **total** | 14 | 100 | 54 | 100 | 203 | 100 | 86 | 100 |  |  |  |  |  |  |
| **sex** |  |  |  |  |  |  |  |  |  |  |  |  |  |  |
| male | - | - | - | - | 74 | 36 | 29 | 33 |  |  |  |  |  |  |
| female | - | - | - | - | 129 | 64 | 57 | 67 | - | - | - | - | - | - |
| **age group** |  |  |  |  |  |  |  |  |  |  |  |  |  |  |
| ≥ 18 and <30 | - | - | - | - | 14 | 7 | 14 | 16 |  |  |  |  |  |  |
| ≥ 30 and <40 | - | - | - | - | 24 | 12 | 12 | 14 |  |  |  |  |  |  |
| ≥ 40 and <50 | - | - | - | - | 41 | 20 | 14 | 16 |  |  |  |  |  |  |
| ≥ 50 and <60 | - | - | - | - | 73 | 36 | 17 | 20 |  |  |  |  |  |  |
| ≥ 60 | - | - | - | - | 51 | 25 | 29 | 34 | - | - | - | - | - | - |
| **raw pork** |  |  |  |  |  |  |  |  |  |  |  |  |  |  |
| no | 5 | 36 | 47 | 87 | 171 | 84 | 70 | 81 |  |  |  |  |  |  |
| yes | 9 | 64 | 7 | 13 | 32 | 16 | 16 | 19 | **12 [3.1-47]** | **< 0.001** | **9.6 [3.0-30]** | **< 0.001** | **7.8 [2.3-26]** | **< 0.001** |
| **raw pork (sausage)** |  |  |  |  |  |  |  |  |  |  |  |  |  |  |
| no | 7 | 50 | 24 | 55 | 136 | 67 | 57 | 66 |  |  |  |  |  |  |
| yes | 7 | 50 | 20 | 45 | 67 | 33 | 29 | 34 | 1.2 [0.36-4.0] | 1.0 | 2.0 [0.68-6.0] | 0.24 | 1.9 [0.63-6.1] | 0.24 |
| **cooked pork** |  |  |  |  |  |  |  |  |  |  |  |  |  |  |
| no | 7 | 50 | 35 | 65 | 115 | 57 | 50 | 58 |  |  |  |  |  |  |
| yes | 7 | 50 | 19 | 35 | 88 | 43 | 36 | 42 | 1.8 [0.56-6.0] | 0.3 | 1.3 [0.44-3.9] | 0.78 | 1.3 [0.44-4.3] | 0.57 |
| **raw beef** |  |  |  |  |  |  |  |  |  |  |  |  |  |  |
| no | 12 | 86 | 52 | 96 | 200 | 98 | 84 | 98 |  |  |  |  |  |  |
| yes | 2 | 14 | 2 | 4 | 3 | 2 | 2 | 2 | 4.3 [0.55-34] | 0.2 | 11 [1.7-73] | < 0.05 | 7 [0.90-54] | 0.093 |
| **raw egg** |  |  |  |  |  |  |  |  |  |  |  |  |  |  |
| no | 13 | 93 | 50 | 93 | 198 | 97 | 83 | 96 |  |  |  |  |  |  |
| yes | 1 | 7 | 4 | 7 | 5 | 3 | 3 | 4 | 0.96 [0.10-9.3] | 1.0 | 3.0 [0.33-28] | 0.33 | 2.1 [0.20-22] | 0.45 |

historical study: case-control study with historical cases and historical controls (2004), HuGO study: case-control study with historical cases and HuGO panel (2019), FM: the frequencies of panel controls are matched to the frequencies of the participants to the 2018 Microcensus of Lower Saxony on sex and age (5 age categories: ≥18 and <30, ≥30 and <40, ≥40 and <50, ≥50 and <60, ≥60), OR: Odds Ratio, 95%CI: 95% Confidence Interval, p: two-sided p-value from Fisher's Exact test, -: not applicable, OR: Odds Ratio, 95%CI: 95% Confidence Interval, p: p-value. Details on the historical study are provided in the Table 2 of the manuscript.

N.B. There is no available information on age and sex for historical cases and controls.

**Table 6. Univariable logistic regression analyses of sex, age and food exposures for gastrointestinal disease in historical and HuGO case-control studies for the investigation of a *Salmonella enterica Oranienburg* outbreak via chocolate, Germany, 2019 (study D).**

|  | historical cases | | historical controls, IM | | panel controls | | panel controls, FM | | historical study | | historical study, IM | | HuGO study | | HuGO study,  FM | |
| --- | --- | --- | --- | --- | --- | --- | --- | --- | --- | --- | --- | --- | --- | --- | --- | --- |
|  | n | % | n | % | n | % | n | % | OR [95%CI] | p | OR [95%CI] | p | OR [95%CI] | p | OR [95%CI] | p |
| total | 48 | 100 | 50 | 100 | 203 | 100 | 148 | 100 |  |  |  |  |  |  |  |  |
| **sex** |  |  |  |  |  |  |  |  |  |  |  |  |  |  |  | |
| male | 24 | 50 | 32 | 64 | 74 | 37 | 74 | 50 |  |  |  |  |  |  |  |  |
| female | 24 | 50 | 18 | 36 | 129 | 63 | 74 | 50 | 1.7 [0.79-3.9] | 0.22 | - | - | 0.57 [0.30-1.1] | 0.10 | - | - |
| **age group** |  |  |  |  |  |  |  |  |  |  |  |  |  |  |  | |
| <10 years old | 22 | 46 | 23 | 46 | 0 | 0 | 0 | 0 |  |  |  |  |  |  |  |  |
| ≥10 years old | 26 | 54 | 27 | 54 | 203 | 100 | 148 | 100 | 1.0 [0.45-2.2] | 1.0 | - | - | - | - | - | - |
| **shopped at the chain x in the last 7 days*** |  |  |  |  |  |  |  |  |  |  |  |  |  |  |  | |
| no | 13 | 29 | 26 | 58 | 174 | 86 | 124 | 84 |  |  |  |  |  |  |  |  |
| yes | 31 | 71 | 19 | 42 | 29 | 14 | 24 | 16 | **3.2 [1.3-7.8]** | **<0.05** | **4.2 [1.2-23]** | **<0.05** | **14 [6.7-30]** | **< 0.001** | **12 [5.6-27]** | **< 0.001** |
| **ate chocolate from chain x in the last 7 days*** |  |  |  |  |  |  |  |  |  |  |  |  |  |  |  | |
| no | 33 | 75 | 43 | 96 | 200 | 98 | 146 | 99 |  |  |  |  |  |  |  |  |
| yes | 11 | 25 | 2 | 4 | 3 | 2 | 2 | 1 | **7.2 [1.5-34]** | **<0.05** | **5.0 [1.1-47]** | **<0.05** | **22 [5.8-83]** | **< 0.001** | **24 [5.1-115]** | **< 0.001** |

historical study: case-control study with historical cases and historical controls (2011), HuGO study: case-control study with historical cases and HuGO panel controls (2019), IM: the controls are individually matched to the cases on sex and age, FM: the frequencies of panel controls are matched to the frequencies of cases on sex, OR: Odds Ratio, 95%CI: 95% Confidence Interval, p: two-sided p-value from Fisher's Exact test, -: not applicable, * missing values for 4/48 historical cases and 5/50 historical controls. Details on the historical study are provided in the Table 2 of the manuscript.

N.B. According to the study authors, none of the other variables were significantly associated with illness (37).
